# Demethylation of leptin promoter in gestational diabetes mellitus: evidence from a mouse model

**DOI:** 10.3389/fcell.2026.1729477

**Published:** 2026-01-21

**Authors:** Linlin Hu, Shihuang Liu, Lin Tu, Shupei Zhang, Xiaojing Huang, Hang Lin, Jianguang Ji, Huan Yi, Xiangqin Zheng

**Affiliations:** 1 Fujian Medical University, Fuzhou, China; 2 Department of obstetrics, The Second Affiliated Hospital of Fujian Medical University, Quanzhou, China; 3 Nan’an Maternal and Child Health Hospital, Quanzhou, China; 4 Department of Gynecologic Oncology, Fujian Maternity and Child Health Hospital, Fujian, China; 5 Faculty of Health Sciences, University of Macau, Taipa, Taipa, Macao SAR China

**Keywords:** gestational diabetes mellitus, glucose metabolism, leptin, methylation, mouse model

## Abstract

**Background:**

Gestational diabetes mellitus (GDM) has been linked to altered leptin (LEP) gene methylation, which may disrupt maternal glucose metabolism and the associated placental signaling. However, the changes of LEP methylation involved in GDM pathophysiology throughout pregnancy remain unclear.

**Methods:**

Female C57BL/6J mice (6–8 weeks old) were randomly divided into control and GDM groups (n = 40 each). The GDM group was fed a high-fat diet for 4 weeks before mating and given a single streptozotocin injection (120 mg/kg, intraperitoneal injection) on gestational day 2, while controls received standard chow and citrate buffer. Fasting blood glucose and body weight were recorded at baseline, gestational days 5, 12, and 18, and postpartum day 1. Oral glucose tolerance tests (OGTTs) were performed at corresponding stages. Blood was collected for measurement of serum leptin concentrations by ELISA. Leptin protein expression and LEP promoter methylation in decidual tissues were analyzed by Western blot and bisulfite pyrosequencing, respectively. Weighted least-squares regression was used to evaluate the associations between leptin, LEP promoter methylation, and glucose metabolism.

**Results:**

The high-fat diet and streptozotocin (HFD + STZ) combination successfully induced a GDM phenotype, as evidenced by early and persistent hyperglycemia and impaired glucose tolerance. Serum leptin levels were significantly increased in GDM mice before pregnancy and returned to the levels of pre-pregnancy in postpartum, indicating that the decidua plays an important role in the dynamic regulation of leptin during pregnancy. Western blot analysis confirmed higher leptin expression in the decidual tissue of GDM mice, while bisulfite pyrosequencing revealed significant demethylation of the LEP promoter. WLS analysis showed that leptin upregulation in GDM was closely associated with epigenetic remodeling at specific CpG sites within the LEP promoter, whereas the relationship between promoter demethylation and FBG was altered in GDM.

**Conclusion:**

Decidual LEP promoter demethylation is associated with hyperleptinemia and shows an epigenetic mechanism linking GDM. LEP promoter demethylation may reflect the metabolic disturbance in GDM and serve as a potential early marker for GDM.

## 1 Introduction

Gestational diabetes mellitus (GDM) is one of the most prevalent metabolic disorders during pregnancy, affecting approximately 10%–20% of pregnancies worldwide and posing significant threats to maternal health (Sweeting et al., 2024). It increases the risk of delivery complications, such as preeclampsia and cesarean section, and has long-term implications for metabolic diseases in both mother and offspring (Sweeting et al., 2024; Bianco and Josefson, 2019; Castaldi et al., 2025). Various predictive approaches have been explored, including maternal anthropometric parameters, biochemical markers, and glucose tolerance tests. However, current methods demonstrate only moderate accuracy (Cooray et al., 2022; Guo et al., 2020), underscoring the need for earlier and more reliable biomarkers for GDM.

Leptin, an adipokine secreted by adipose tissue and placenta, plays a pivotal role in glucose homeostasis and insulin sensitivity (Dagogo-Jack, 2024; Munzberg et al., 2024; Mallardo et al., 2021). Clinical studies have consistently reported elevated maternal serum leptin levels in early and mid-pregnancy among women with GDM (Qiu et al., 2004). Notably, maternal leptin levels correlate positively with insulin resistance and fasting glucose levels (Hosseini et al., 2023), underscoring its role in glucose metabolic interplay. Consistently, prior studies have shown that demethylation of the leptin (LEP) promoter in placental tissue is inversely correlated with leptin mRNA expression (Hogg et al., 2013), supporting a mechanistic link between promoter demethylation and aberrant leptin signaling in GDM. However, most existing studies remain limited to cross-sectional designs and have not addressed the temporal dynamics of leptin promoter methylation throughout pregnancy. It also remains unclear whether integrating LEP promoter demethylation could further improve the predictive performance of leptin-related markers for GDM.

Therefore, we hypothesized that Demethylation of the LEP promoter in decidual tissue is a major cause of hyperleptinemia in GDM, which subsequently contributes to maternal glucose metabolic disturbances. To test this hypothesis, we established a high-fat diet combined with streptozotocin (HFD + STZ)-induced GDM mouse model that closely reproduces the clinical dynamic features. We systematically evaluated maternal blood glucose, serum and tissue leptin levels, and the methylation status of the leptin promoter in decidual tissue to investigate the epigenetic mechanism underlying leptin elevation in GDM and explore its potential as an early biomarker for predicting glucose metabolism abnormalities.

## 2 Methods

### 2.1 Animal model and experimental design

Female C57BL/6J mice (6–8 weeks old, 18–22 g) were obtained from an accredited vendor and housed under specific pathogen-free (SPF) conditions with a 12 h light/dark cycle, temperature of 22 °C ± 2 °C, and humidity of 50% ± 10%. After 1 week of acclimatization, mice were randomly assigned to either the GDM model group or the control group (n = 40 per group). The GDM group was fed a high-fat diet (60% energy from fat, HFD) for 4 weeks prior to mating and received a single intraperitoneal injection of streptozotocin (STZ, 120 mg/kg) on gestational day 2 (GD2). Control mice were maintained on standard chow and injected with an equivalent volume of citrate buffer.

Pregnancy was confirmed by the presence of a vaginal plug (designated GD0) and serum progesterone measurement on GD5. Fasting blood glucose (FBG) were monitored at baseline, GD5, GD12, GD18, and postpartum day 1 (PP1). Oral glucose tolerance tests (OGTTs) were performed at designated gestational stages. At each time point, subsets of 6 dams per group were sacrificed for sample collection.

All animal procedures were approved by the Institutional Animal Care and Use Committee of Fujian Maternity and Child Health Hospital (Approval No.: AEC SFY 2025 022).

### 2.2 Sample allocation and collection schedule

To ensure adequate samples for both longitudinal serum assays and stage-specific tissue analyses, fixed subsets of mice were pre-assigned to scheduled sacrifices as follows: 1)Pre-pregnancy baseline (PreP): 6 per group; 2)After 4 weeks of premating HFD: 6 per group; 3)GD5, GD12, GD18, PP1: 6 per group at each time point. A total of 36 mice per group were designated for tissue collection. The remaining ∼4 animals per group were retained as backups to account for potential pregnancy failure or unexpected mortality. Serum sampling was performed longitudinally in all surviving dams at each time point.

### 2.3 Blood and tissue collection

Blood was collected via retro-orbital venous plexus under light anesthesia. Serum was isolated by centrifugation at 3,000 rpm for 10 min and stored at −80 °C. Decidual tissue were rapidly dissected, rinsed in cold PBS, and divided into aliquots for snap-freezing in liquid nitrogen for molecular assays.

### 2.4 Biochemical assays

Serum leptin concentrations were quantified by enzyme-linked immunosorbent assay (ELISA, E-EL-M3008, Elabscience, China) according to the manufacturer’s instructions. Western blotting was performed on decidual lysates to determine leptin (ab16227, Abcam, United States) protein expression, normalized to β-actin.

### 2.5 DNA methylation analysis

**FIGURE 1:**
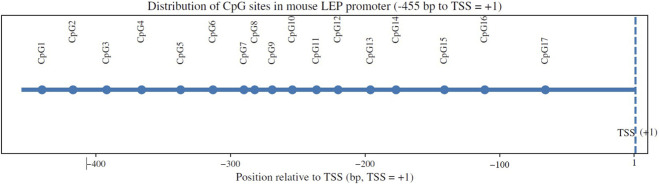
Schematic distribution of CpG sites within the mouse LEP promoter region. A schematic diagram illustrating the approximate locations of 17 CpG sites (CpG1–CpG17) within the LEP promoter region, shown relative to the transcription start site. CpG positions are indicated in base pairs (bp) along the promoter sequence.

Genomic DNA was extracted from decidual tissues using a commercial DNA extraction kit (DP304, TIANGEN, China). Bisulfite conversion was carried out using the EZ DNA Methylation Kit (D0068M, Beyotime, China). The CpG-rich region upstream of the leptin transcription start site was amplified by PCR, and methylation levels at individual CpG sites were quantified by pyrosequencing (QuantStudio 3, ThermoFisher, USA). Methylation percentages were calculated as the proportion of cytosines remaining unconverted at each CpG site. Methylation rate (%) = M/(M + U) × 100% (M and U are signal intensities amplified by methylated and unmethylated primers, respectively). Methylation primers were designed based on the CpG island in the promoter region of the mouse Leptin gene, covering multiple CpG sites (Figure 1; Supplementary Table S1).

### 2.6 Statistical analysis

All quantitative data are expressed as mean ± standard deviation (SD). Group comparisons were performed using Student’s t-test or analysis of variance (ANOVA) with Bonferroni correction as appropriate. Associations between leptin, LEP promoter methylation, and glucose metabolism were evaluated using weighted least-squares (WLS) regression. Analyses were performed on group mean values (mean ± SEM) across five gestational time points, applying weights of N/SD^2^ to account for variability in measurement precision. The WLS estimator was calculated as β̂ = (X^T^WX)^−1^X^T^Wy, where W is a diagonal matrix of weights (N/SD^2^), giving greater influence to estimates with lower variance. Model assumptions were verified through residual analysis, and heteroscedasticity was corrected using WLS weighting. Regression outputs included slope coefficients (β_1_), coefficient of determination (R^2^), two-sided P values, and 95% confidence intervals. For multiple CpG site comparisons, P values were adjusted using the Benjamini–Hochberg false discovery rate (FDR) method. Statistical analyses were conducted in SPSS 26.0 (IBM, United States) and GraphPad Prism 9 (GraphPad Software, United States).

## 3 Results

### 3.1 Establishment of the GDM mouse model

**FIGURE 2:**
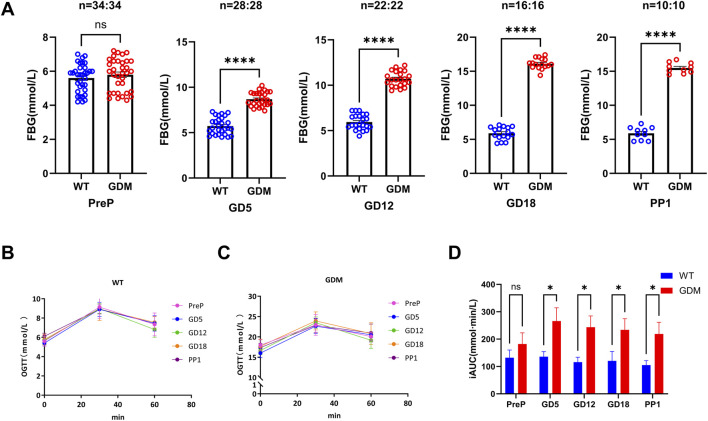
Establishment of the gestational diabetes mellitus (GDM) mouse model. **(A)** Fasting blood glucose (FBG) levels in control (WT) and GDM mice at pre-pregnancy (PreP), gestational days 5, 12, and 18 (GD5, GD12, GD18), and postpartum day 1 (PP1). GDM mice developed progressive hyperglycemia from GD5 onward (****P < 0.0001). **(B,C)** Oral glucose tolerance tests (OGTT) for WT and GDM mice at different gestational stages, demonstrating impaired glucose clearance in the GDM group [Sample sizes for each period are the same as **(A)**]. **(D)** Incremental area under the curve (iAUC) of OGTT, showing a significant increase in glucose intolerance across gestation in GDM mice (P < 0.05). Data are presented as mean ± SD. ns, not significant; P < 0.05, *P < 0.05, **P < 0.01, ****P < 0.0001.

The combination of HFD and STZ successfully induced GDM-like phenotypes in pregnant mice. Fasting blood glucose (FBG) levels in the GDM group were elevated as early as GD5 (7.58 ± 1.91 mmol/L vs. 6.17 ± 0.89 mmol/L, P < 0.05). FBG continued to rise throughout gestation, peaking at GD18 (16.12 ± 1.82 mmol/L), and remained significantly higher than controls on postpartum day 1 (14.24 ± 2.32 mmol/L, P < 0.01) (Figure 2A). Oral glucose tolerance tests (OGTT) demonstrated impaired glucose clearance in GDM mice, as indicated by significantly higher incremental area under the curve (iAUC) values compared with controls (P < 0.01) (Figures 2B–D).

### 3.2 Serum leptin levels during pregnancy

**FIGURE 3:**
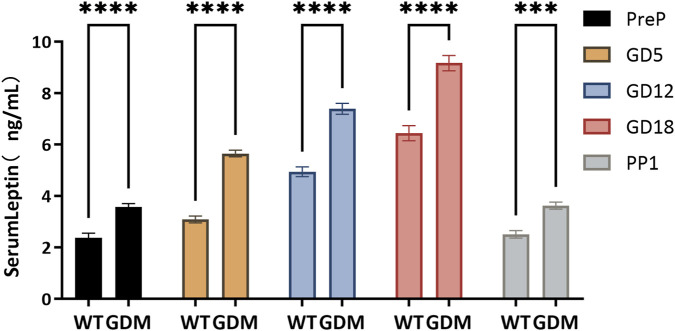
Serum leptin concentrations during pregnancy in control (WT) and GDM mice. Serum leptin levels were measured at pre-pregnancy (PreP), gestational days 5, 12, and 18 (GD5, GD12, GD18), and postpartum day 1 (PP1). Sample sizes as follows: PreP (n = 34 per group), GD5 (n = 28 per group), GD12 (n = 22 per group), GD18 (n = 16 per group), and PP1 (n = 10 per group). Leptin concentrations increased progressively during gestation in both groups but were consistently higher in GDM mice. Significant differences were observed from GD5 onward, with peak levels at GD18. Data are expressed as mean ± SD. ns, not significant; ***P < 0.001; ****P < 0.0001.

Serum leptin concentrations increased progressively during gestation in both groups but were consistently higher in the GDM mice. On gestational day (GD) 5, serum leptin in the GDM group was (5.41 ± 0.47) ng/mL, significantly higher than the control group (4.62 ± 0.53) ng/mL (P < 0.05). This trend persisted through GD12 [(6.21 ± 0.52) vs. (5.48 ± 0.57) ng/mL, P < 0.01] and GD18 [(7.94 ± 0.63) vs. (6.65 ± 0.97) ng/mL, P < 0.01], as illustrated in Figure 3. Notably, by postpartum day 1, serum leptin levels in both groups had returned to values comparable to those measured before pregnancy, suggesting rapid normalization after delivery.

### 3.3 Leptin protein expression in decidual tissues

**FIGURE 4:**
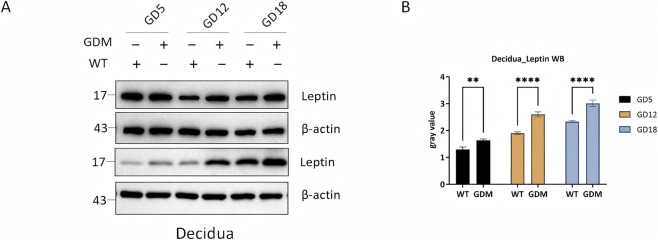
Leptin protein expression in decidual tissues of control (WT) and GDM mice. **(A)** Representative Western blots showing leptin and β-actin expression in decidual tissues from WT and GDM mice at gestational days 5, 12, and 18 (GD5, GD12, GD18). **(B)** = Quantification of decidual leptin protein levels demonstrating progressive increases during pregnancy and markedly elevated expression in GDM mice compared with controls. Decidual tissues were collected from n = 6 mice per group at each time point. Data are presented as mean ± SD. **P < 0.01, ****P < 0.0001.

Western blot analysis showed that leptin expression in decidual tissue was markedly higher in GDM mice, with the most pronounced differences observed in mid- and late gestation (P < 0.0001) (Figures 4A,B).

These findings parallel the serum leptin profile, reinforcing that GDM enhances both systemic leptin levels and local leptin production within the decidua.

### 3.4 Methylation level of the leptin promoter

**FIGURE 5:**
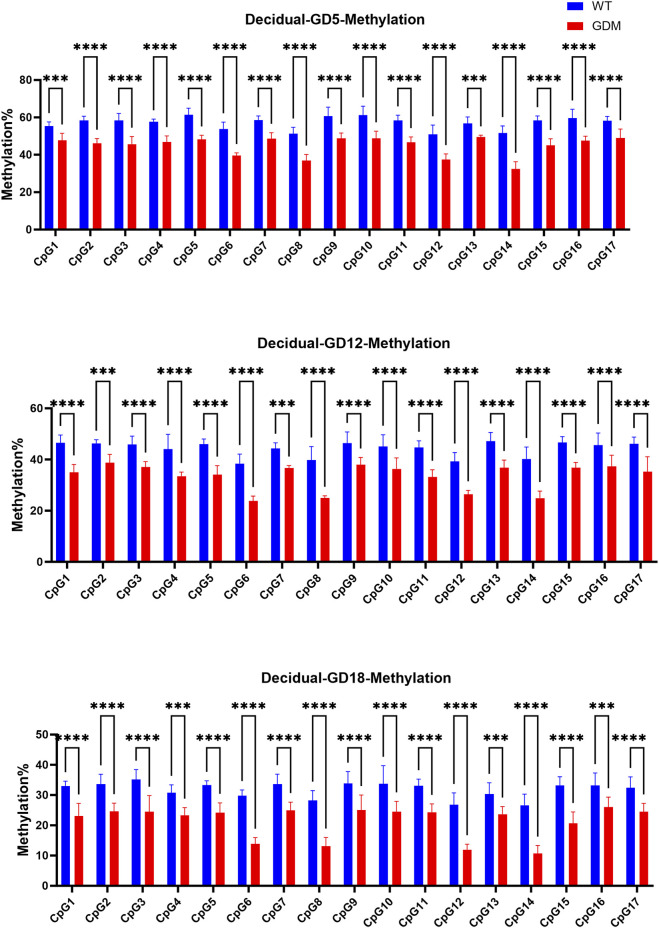
Methylation levels of the leptin (LEP) promoter in decidual tissues of WT and GDM mice. Methylation percentages of the same 17 CpG sites in decidual tissue at GD5, GD12, and GD18. GDM mice exhibited marked hypomethylation across multiple CpG sites compared with controls (P < 0.01 to **P < 0.0001), indicating tissue-specific demethylation of the LEP promoter during gestation. Decidual tissues were collected from n = 6 mice per group at each time point. Data are expressed as mean ± SD. ***P < 0.001, ****P < 0.0001, ns, not significant.

The bisulfite pyrosequencing analysis of the LEP promoter revealed significantly reduced methylation levels in decidual tissue of GDM mice compared with controls. The average methylation percentage in GDM mice was (41.23 ± 3.51)%, significantly lower than that in controls (54.47 ± 4.26)% (P < 0.001). (Figure 5). These findings further demonstrated that the increased leptin levels in the decidua of GDM mice were associated with demethylation at multiple CpG sites within the LEP promoter, suggesting that epigenetic remodeling may contributes to enhanced leptin synthesis in maternal decidual tissue.

### 3.5 Relationship between leptin, LEP methylation, and glucose metabolic changes

Weighted regression analysis again showed that serum leptin exhibited only weak and nonsignificant associations with fasting glucose in both groups and similarly weak associations with OGTT-derived iAUC, indicating that leptin alone accounted for limited variation in maternal glycemic indices.

**FIGURE 6:**
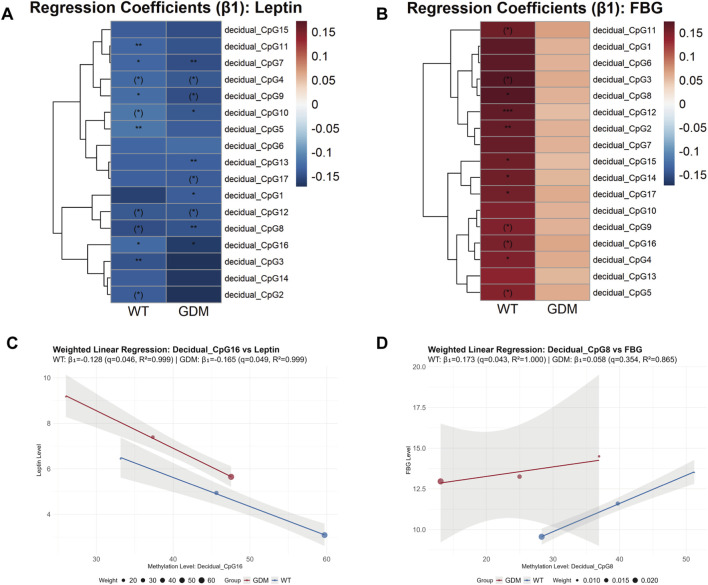
Weighted regression analysis linking LEP promoter methylation to leptin levels and fasting blood glucose (FBG) in WT and GDM mice. **(A,B)** Heatmaps showing weighted regression coefficients (β_1_) for individual CpG sites within the leptin (LEP) promoter in relation to serum leptin levels **(A)** and FBG **(B)** in WT and GDM mice. CpG methylation levels were quantified in decidual tissues collected from n = 6 mice per group at each gestational time point. CpG sites marked with brackets indicate associations that were statistically significant before false discovery rate (FDR) correction but did not remain significant after correction, whereas CpG sites without brackets retained statistical significance following correction (P/q < 0.05, *P/q < 0.01, **P/q < 0.001). **(C,D)** Representative weighted linear regression plots illustrating associations between LEP promoter methylation and serum leptin levels **(C)** or FBG **(D)** for top1 CpG sites. Regression analyses were performed using group-level observations across gestational stages, with weights defined as N/SD^2^, where N denotes the number of animals contributing longitudinal serum measurements at each time point. Serum leptin and glucose data were obtained longitudinally, with sample sizes of 28, 22, and 16 animals per group at GD5, GD12, and GD18, respectively. Data are presented as group means.

At the epigenetic level, introducing multivariable adjustment revealed clearer CpG-specific patterns. Prior to multiple-test correction, several CpG sites displayed significant associations with leptin or FBG. After FDR correction, the CpG sites significantly associated with serum leptin levels differed between the two groups (Figures 6A,C), with the exception of CpG7 (WT group: β1 = −0.133, 95%CI: −0.170 to −0.097, q = 0.046; GDM group: β_1_ = −0.148, 95%CI: −0.162 to −0.134, q = 0.026) and CpG16 (WT group: β_1_ = −0.128, 95%CI: 0.169 to −0.087, q = 0.046; GDM group: β_1_ = −0.165, 95%CI: 0.216 to −0.114, q = 0.049), which were significant in both WT and GDM mice. In contrast, for FBG, CpG sites with FDR-adjusted statistical significance were identified only in the WT group (Figures 6B,D). No CpG sites showed statistically significant associations with OGTT-derived indices in either group after FDR adjustment.

## 4 Discussion

In this study, we established a reproducible GDM mouse model using a combination of HFD + STZ, which partially mimicked key clinical features of GDM, including maternal hyperglycemia and elevated leptin levels (Pandey et al., 2023; Gao et al., 2021). This classical induction method captures important aspects of the dynamic clinical course of human GDM, characterized by progressive glucose intolerance during gestation. Our findings highlight several key experimental results, including early-onset and sustained hyperglycemia and impaired glucose tolerance, serum leptin differences evident before pregnancy, widening across gestation and returning rapidly to pre-pregnancy levels postpartum, increased leptin expression and LEP promoter demethylation in decidual tissue, and stronger correlations between specific CpG demethylation events and FBG than those observed for serum leptin levels, which may offer additional insight into how decidual LEP promoter methylation relates to maternal metabolic dysregulation in GDM. Unlike previous studies primarily focusing on placental LEP methylation in human cohorts, our work characterizes time-resolved LEP promoter methylation in maternal decidual tissue within a GDM mouse model, allowing a more detailed comparison between circulating leptin, tissue-specific epigenetic changes and FBG.

### 4.1 Dynamic changes in leptin and GDM pathophysiological implications

In our study, the progressive elevation of leptin observed throughout gestation in GDM mice indicates a state of chronic hyperleptinemia. This finding aligns with clinical observations that elevated leptin precedes or parallels glucose intolerance in pregnant women (Qiu et al., 2004; Bawah et al., 2019; Jeon et al., 2017). Compared with glucose metabolism indicators in Figure 2, the significance in leptin levels appeared earlier than changes in FBG and OGTT results, even showing differences before pregnancy. This suggests that this pattern raises the possibility that leptin might serve as an earlier indicator of glucose dysregulation in GDM. If confirmed in human studies, such early changes could help to define a window for preventive interventions. Previous clinical studies reporting the predictive value of leptin for GDM are consistent with our findings (Qiu et al., 2004; Liu and Wang, 2025), supporting its potential utility as an early biomarker. Moreover, serum leptin levels rapidly returned to pre-pregnancy levels by postpartum day 1, suggesting that decidual leptin synthesis and secretion may contribute to maintaining circulating leptin during pregnancy. The exaggerated rise in leptin during mid-to late pregnancy (Figure 3) may reflect both increased placental synthesis and the development of leptin resistance, as suggested by previous human and animal studies, which have implicated leptin signaling in maternal insulin resistance and energy storage. Enhanced leptin protein expression in decidual tissue further suggests a potential role for local leptin signaling in modulating nutrient transport and immune responses at the maternal-fetal interface, consistent with its proposed role in metabolic adaptation to pregnancy (Lepercq et al., 1998; Hoggard et al., 2001; Perez-Perez et al., 2018).

### 4.2 Leptin promoter demethylation and epigenetic regulation

Building upon the observed increase in decidual leptin expression, we next investigated the underlying epigenetic mechanism. One of the key findings of this study is the tissue-specific demethylation of the LEP promoter in decidual tissue of GDM mice (Figure 5). This demethylation, particularly evident at the CpG16 site, showed the strongest model fit (R^2^ = 0.999) and statistical significance (WT: β_1_ = −0.128, p = 0.016; GDM: β_1_ = −0.165, p = 0.015), indicating that reduced methylation at this locus is strongly associated with higher leptin production in decidual tissue. These findings support the hypothesis that decidual LEP promoter demethylation may contribute to leptin overproduction and may represent one of the epigenetic mechanisms involved in maternal metabolic adaptation to GDM. Recent research has shown that maternal environmental factors during pregnancy, such as high-fat diet and hyperglycemia, can remodel gene expression across different tissues through epigenetic and metabolic pathways. Multiple studies have demonstrated that the LEP promoter in placental tissue from women with GDM is significantly hypomethylated and inversely correlated with leptin mRNA expression (Melzner et al., 2002; Marchi et al., 2011). Moreover, adverse maternal conditions—including high-fat diet, obesity, and hyperglycemia—can induce LEP promoter hypomethylation (Xia et al., 2014; Wieting et al., 2019), leading to leptin overexpression and aggravation of insulin resistance and glucose dysregulation (Sletner et al., 2021; Mosher et al., 2016). Our data extend these observations by showing analogous LEP promoter hypomethylation in maternal decidual tissue in a time-resolved GDM mouse model, linking tissue-specific epigenetic changes to both circulating leptin and dynamic measures of glucose metabolism.

### 4.3 Correlation between leptin or LEP promoter demethylation and glucose metabolism

To further validate the relationship between leptin and glucose dysregulation, we conducted an integrated correlation analysis. The results showed that CpG sites of LEP promoter demethylation were associated with serum leptin and FBG, though the strength of association differed. Weighted regression analysis indicated that serum leptin showed only modest correlations with FBG and OGTT iAUC, suggesting that circulating leptin levels alone may not fully account for the observed glucose dysregulation. This is generally consistent with findings from previous studies (Sengul et al., 2009; D'Souza et al., 2017; Wang et al., 2019). In contrast, methylation at specific CpG sites, particularly CpG8 and CpG12 for FBG, showed stronger correlations. These findings raise the possibility that methylation-based indicators might be more closely associated with FBG in GDM than serum leptin levels, and could therefore be explored as candidate epigenetic markers in future studies. Previous bioinformatics studies have attempted to identify gene sets and corresponding CpG sites to predict GDM and related complications, but their predictive performance has been limited (Li et al., 2019; Liu et al., 2021), likely due to the absence of physiologically grounded molecular validation. In contrast, our study focuses on leptin, as an entry point to explore LEP promoter methylation changes, thereby illustrating a biologically grounded approach to epigenetic biomarker exploration.

Conversely, when comparing the correlations of individual demethylated CpG sites, we found that the CpG sites associated with serum leptin levels were not fully overlapping between the WT and GDM groups (Figure 6A). This observation may indicate that the epigenetic regulation of decidual (placental) leptin production via LEP promoter demethylation differs between physiological and GDM conditions. With respect to FBG, associations with CpG demethylation were observed only in the WT group (Figure 6B), whereas no statistically significant correlations were detected in the GDM group. From this perspective, under physiological conditions, decidual (placental) leptin production appears to be associated with glycemic status, while this association seems to be attenuated or altered in GDM.

### 4.4 Study limitations and future perspectives

This study has certain limitations. The sample size at each time point was relatively small; therefore, we applied weighted least squares (WLS) regression with sample size-based weighting and used standard error of the mean (SEM) calculations to minimize variance and improve reliability. Interspecies differences also limit direct extrapolation to humans. Moreover, we did not investigate downstream signaling pathways, such as JAK/STAT3 or PI3K/AKT, that may mediate leptin’s metabolic effects (Trujillo-Guiza and Senaris, 2018). Future research will likely benefit from multi-omics approaches integrating methylome, transcriptome, and proteome profiling to delineate the broader regulatory network influenced by leptin demethylation. Longitudinal studies in human cohorts are also needed to clarify whether LEP promoter demethylation precedes the onset of GDM and can serve as a predictive biomarker. Furthermore, the long-term glucose recovery profile of this GDM model will be incorporated into monitoring protocols, thereby providing a theoretical basis for evaluating and developing more suitable animal models of GDM.

## 5 Conclusion

The LEP promoter in decidual tissue exhibited significant demethylation that was associated with serum leptin levels. The altered association between LEP promoter methylation and FBG in GDM suggests a decoupling of epigenetic leptin activation from glucose regulation, which may have implications for metabolic risk stratification. These results provide experimental evidence for its potential role in GDM early diagnosis and prevention.

## Data Availability

The original contributions presented in the study are included in the article/Supplementary Material, further inquiries can be directed to the corresponding authors.
